# Phytochemical and Pharmacological Review of* Cryptolepis sanguinolenta* (Lindl.) Schlechter

**DOI:** 10.1155/2017/3026370

**Published:** 2017-10-15

**Authors:** Newman Osafo, Kwesi Boadu Mensah, Oduro Kofi Yeboah

**Affiliations:** Department of Pharmacology, Faculty of Pharmacy and Pharmaceutical Sciences, College of Health Sciences, Kwame Nkrumah University of Science and Technology, Kumasi, Ghana

## Abstract

**Ethnopharmacological Relevance:**

* Cryptolepis sanguinolenta* is a scrambling thin-stemmed shrub found in Africa. Traditionally in West Africa, it is employed in the treatment of malaria, diarrhea, and respiratory conditions. This review discusses the traditional importance as well as the phytochemical, ethnomedical, pharmacological, and toxicological importance of this plant.

**Materials and Methods:**

Excerpta Medica Database, Google Scholar, Springer, and PubMed Central were the electronic databases used to search for and filter primary studies on* Cryptolepis sanguinolenta*.

**Results:**

The detailed review of various studies conducted on* C. sanguinolenta* and some of its constituents gives an important body of proof of its potential therapeutic benefits and also of its use as a source of lead compounds with therapeutic potentials.

**Conclusion:**

The review on* C. sanguinolenta* is important in identifying grey areas in the research on this medicinal plant and also provides comprehensive data thus far to continue research on this plant.

## 1. Introduction

 The African-indigenous, scrambling thin-stemmed shrub,* Cryptolepis sanguinolenta* (Lindl.) Schlechter (Apocynaceae), is a plant of interest in West African ethnomedicine. Traditionally, the shrub is used in the treatment of various diseases such as malaria, bacterial respiratory diseases, hypertension, and diarrhea and as a cicatrizant [1–4]. The plant has received a lot of research attention for decades, leading to the identification and characterization of certain secondary metabolites, some of which have served as lead compounds in drug development processes [5–9]. These studies have also led to the establishment of several pharmacological activities of the plant, including anti-inflammatory, anticancer, antidiabetic, antithrombotic, antihypertensive, and antipyrexic effects [10–12]. However, no recent review exists on* C. sanguinolenta,* combining and detailing the phytochemical composition, the pharmacological activities, and the reported pharmacokinetic and toxicological studies, which has called for the need to compile all available data on* C. sanguinolenta*. This review therefore seeks to highlight the folkloric significance, phytochemical composition, and biological and pharmacological activities of* C. sanguinolenta*. This review will also aid future studies aimed at isolation, purification, and characterization of the various bioactive compounds responsible for the reported biological and pharmacological activities of this plant.

## 2. Ethnopharmacological and Other Local Uses

*Cryptolepis sanguinolenta* is widely used in the West African community and other localities in the management of various ailments, as summarized in Table 1. Its ethnomedicinal popularity has warranted the various activities of the plant to be documented in several research publications. The length of its branches and its twining and scrambling nature enable its use as a rope in the construction of houses. The pulverized root can also be used as a dye [13, 14].

## 3. Taxonomy and Local Names

*Cryptolepis sanguinolenta* belongs to kingdom Plantae, phylum Magnoliophyta, class Equisetopsida. It is in the subclass Magnoliidae and superorder Asteranae. The plant belongs to order Gentianales, family Apocynaceae, subfamily Periplocoideae. This plant belongs to the genus* Cryptolepis* and the species* sanguinolenta* [28, 29].  Table 2 contains the various local names by which the plant is referred to in different localities.

## 4. Ecology

*Cryptolepis sanguinolenta* is native to West Africa and is commonly found in tropical rainforests, thickets, and mountainous ecologies [21, 36]. In Ghana, it is mostly found in the month of June in areas of the Akwapim and Aburi mountains where there is enough rainfall [21, 33, 36]. The plant thrives well in the woody savannah and areas with adequate sunlight and water and, as such, can also grow in certain areas of Central Africa, specifically Congo-Brazzaville, but is completely absent in mushy and salty swamps of coastal regions [31, 33]. However, in certain areas such as around Lake Bosomtwe, in the Ashanti Region of Ghana, the plant flourishes in April and is sometimes the main vegetation cover on deserted farmlands [31, 37].* Cryptolepis sanguinolenta* may also occur in dry and gallery forests, usually near water, from sea level up to 850 m altitude [13].

## 5. Plant Description

**Figure 1:**
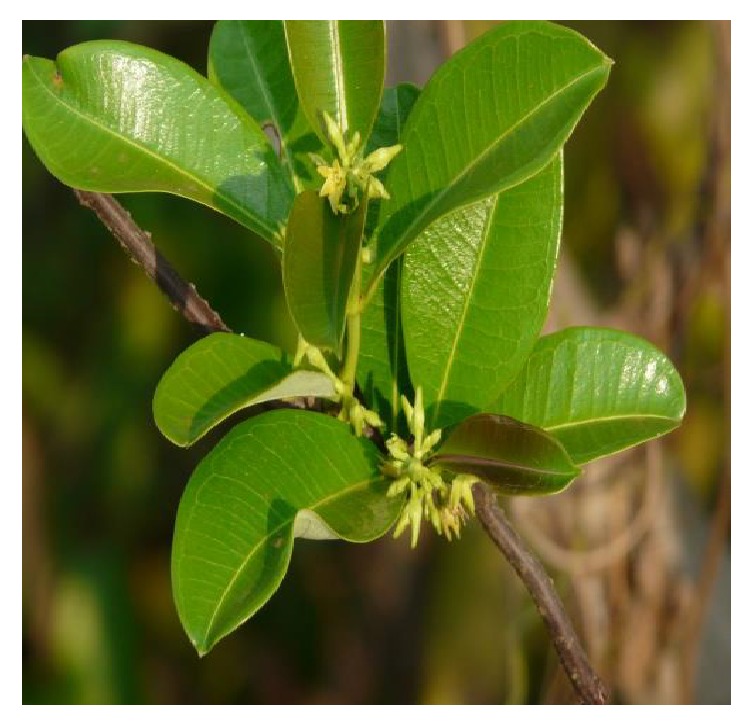
The leaves and flowers of* Cryptolepis sanguinolenta* (adapted from the Useful Tropical Plants Database, 2014).

*Cryptolepis sanguinolenta* is a thin-stemmed twining and scrambling shrub with orange-colored sap in the cut stem which becomes red on ripening [7, 33, 38]. The leaves (Figure 1) are opposite, simple, and entire with an acute to shortly acuminate apex and a symmetrical base. The plant has cymose inflorescence up to 8 cm. The leaves are petiolate, glabrous, and elliptic to oblong-elliptic, up to 7 cm long and 3 cm wide. The inflorescence cymes, lateral on branch shoots, are few flowered, with a yellow corolla tube up to 5 mm long [13, 17, 18, 33]. Flowers are bisexual, regular, 5-merous, 1.5 cm long, and greenish yellow with a pedicel 0.5–1.5 cm long and sepals lanceolate and acute. The corolla is 5 mm long with star-like arranged lanceolate, 12 mm long and contorted to the left in bud. The fruits are paired in linear follicles and are horn-like. The seeds are oblong in shape, small and pinkish, 10–12 mm long, and embedded in a tuft of silky hairs [31, 33]. The dried leaves, stems, and roots have a sweet fragrance. The root varies from 0.4 to 6.6 cm in length and from 0.31 to 1.4 cm in width and has a bitter taste. The roots are rather tortuous and branched with little or no rootlets. The outer surface is yellowish brown and when dry shows longitudinal ridges with occasional cracks. The roots break easily with fractures leaving a smooth transverse surface, which is yellow in color. The sap is extremely bitter and is characterized by the rapidity with which it turns deep red on exposure to air [13, 18, 39, 40].

## 6. Morphoanatomical and Histological Evaluation

Pharmacognostic evaluation of plants is critical in the selection of plants so as to avoid fatalities associated with the use of inappropriate plant materials in herbal preparations. Morphoanatomical and histological profiles of plants may also be useful in the standardization and in the identification of adulterants in plant materials or herbal preparations. Ameyaw [31], in his analysis of the morphological characters of* C. sanguinolenta* from three different locations, identified that the shrub harvested from these different locations showed statistically significant (*p* < 0.001, *p* < 0.01, and *p* < 0.05) variability, which he attributed to changes in edaphic and environmental factors such as soil characteristics. Morphoanatomical analysis included the leaf, pod, and seed indices and the leaf petioles (Table 3). The root's histological features studied included the fiber index, vessel element index, and prismatic crystal index (Table 4).

## 7. Phytochemical Composition

**Figure 2:**
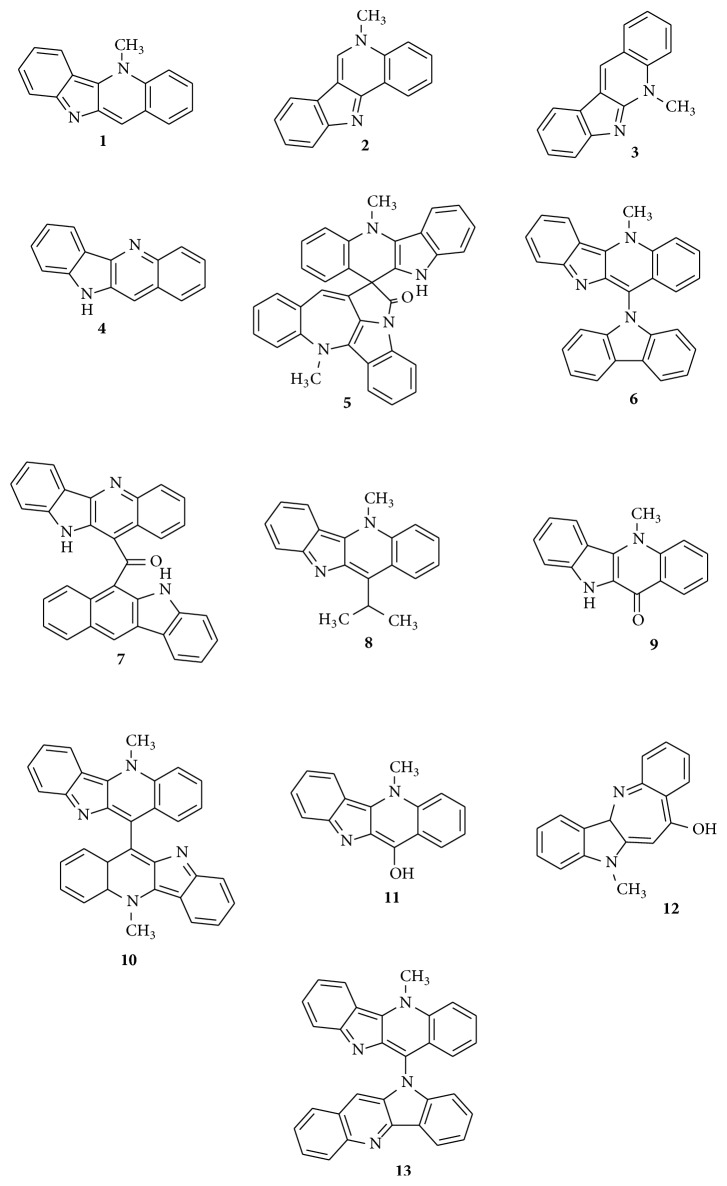
Structures of alkaloids isolated from the root of* Cryptolepis sanguinolenta*.

Phytochemical analysis of various fractions of* C. sanguinolenta* has shown the presence of a variety of secondary metabolites. The methanol extract of the plant has been shown to contain alkaloids, tannins, and flavones [26, 41]. Unlike the analysis performed by Tona et al. [20], phytochemical screening by Claude et al. [26] did not indicate the presence of flavonoids in the root bark, probably due to the difference in the methods employed in the preparation of the plant material. Studies by Mills-Robertson et al. [42, 43] revealed the presence of reducing sugars, polyuronides, alkaloids, and anthocyanosides in the ethanol, water, and chloroform extracts with the water fraction containing triterpenes in addition. The major alkaloid in* C. sanguinolenta* root is the indoloquinoline cryptolepine** (1)** which was first isolated in 1931 and has since been reported to be present in the plant from Ghana and Nigeria [5, 16, 44]. Extensive research on cryptolepine has shown its anti-inflammatory, hypotensive, antithrombotic, antidiabetic, antibacterial, antiplasmodial, antipyretic, and renovascular vasodilatory effects [6, 45–47]. Two research groups, Pousset et al. [48] and Sharaf et al. [49, 50], independently reported related alkaloids and named them isocryptolepine** (2)** and cryptosanguinolentine, respectively. Isocryptolepine is an angularly fused alkaloid with an indolo[3,2-c]quinoline ring system, whereas cryptolepine is a linearly fused alkaloid with an indolo[3,2-b]quinoline ring system [51]. Subsequently, Cimanga et al.'s group [52] and Sharaf et al.'s group [49], in independent studies, reported new linearly fused indolo[2,3-b]quinoline alkaloids and named them neocryptolepine** (3)** and cryptotackieine, respectively. Other alkaloids reported from the plant* C. sanguinolenta* include quindoline** (4)**, the spirononacyclic alkaloid cryptospirolepine** (5)**, cryptolepicarboline** (6)**, cryptomisrine** (7)**, 11-isopropylcryptolepine** (8)**, cryptolepinone** (9)**, and biscryptolepine** (10)**. Paulo et al. [53] and Crouch et al. [54] also reported other isolated indole alkaloids including hydroxycryptolepine** (11)**, cryptoheptine** (12)**, and cryptoquindoline** (13)** (Figure 2).

## 8. Analytical Techniques

Various analytical procedures, qualitative and quantitative, have been employed in the analysis of the phytochemical composition of* C. sanguinolenta,* mostly its alkaloidal content. Ameyaw and Duker-Eshun employed the N/10 iodine solution and Mayer's and Dragendorff's reagents to confirm the presence of alkaloids in a hydroalcoholic extract of the plant [41]. Similarly, Tona and his team also employed Dragendorff's reagent in the analysis of the plant alkaloids [20]. In a quantitative analysis of the alkaloidal content by Ameyaw and Duker-Eshun [41], the chloroform extract of the plant alkaloids was dried with anhydrous sodium sulphate, and the dry residue was weighed and its percentage was determined from the weight of the plant material used.

Paulo and his team [53] performed ^1^H- and ^13^C-NMR analysis of cryptolepine, quindoline, hydroxycryptolepine, cryptoheptine, and cryptoquinoline isolated from* C. sanguinolenta*. Following extraction and isolation of the alkaloidal extract of the plant material, the team performed fractionation by thin-layer chromatography on an acid alumina column to yield five fractions. Subsequent purification of the fractions by preparative thin-layer chromatography (TLC) on silica gel developed with CHCl_3_-MeOH at various percentages yielded quindoline and hydroxycryptolepine. Grellier et al. [55] also isolated quindoline and isocryptolepine but not hydroxycryptolepine by chromatographing over silica and subsequent elution with hexane and then with CH_2_Cl_2_. Isocryptolepine was separated by preparative silica gel thin-layer chromatography. Column chromatography (CC) of a second fraction, on a neutral alumina column with EtOAc-MeOH, led to the isolation of cryptoquindoline, cryptolepine, isocryptolepine, and neocryptolepine following respective developing techniques. Purification of another fraction by preparative TLC on silica gel, developed with an ammoniacal chloroform-methanol solvent system, yielded cryptoheptine. Paulo and his colleagues investigated the natural occurrence of cryptoquindoline by TLC-densitometry and ultraviolet spectrophotometry [53].

Tona et al. [20], after examining individual alkaloids in the alkaloid fraction obtained by a classical acid : base extraction procedure for alkaloids, analyzed the alkaloids by TLC in an ammoniacal chloroform-methanol solution similar to the method employed by Paulo et al. but with slight variation in the individual solvent proportion. TLC spots were developed by spraying with Dragendorff's reagent [20, 53]. To detect flavonoids, TLC was developed in an aqueous alcohol-acid solvent system and the spots were visualized with 1% aluminium chloride solution in methanol under UV 366 nm as described by Harborne. Tannins were identified using 1% gelatin solution, saponins by froth test, and anthraquinones with 10% potassium hydroxide solution in methanol [20, 56, 57]. Tona et al. [20] used hexane : ethyl acetate mobile phase system and Liebermann-Burchard as a reagent to analyze terpenes and sterols.

Paulo et al. [53] studied the alkaloidal content of the leaf and root aqueous extract of* C. sanguinolenta* by comparing the chromatograms of extracts with those of isolated alkaloids using HPTLC in situ UV technique. Another analytical technique used by the team is recording infrared spectra in potassium bromide on a PerkinElmer 1420. The group obtained laser desorption ionization mass spectra (LDI-MS) with a 2,5-dihydroxybenzoic acid matrix using a time-of-flight instrument. In addition to the ^1^H- and ^13^C-NMR spectroanalysis performed in their earlier study, high-performance thin-layer chromatography was performed [53].

## 9. Pharmacological Activity

### 9.1. Antimalarial

*Cryptolepis sanguinolenta* is widely used in the Ghanaian community for the management of uncomplicated malaria. Komlaga et al. [58], in the analysis of the composition of various antimalarial herbal preparations, identified that the majority of the study preparations (47.8%) contain* C. sanguinolenta*. Among natural products, indole alkaloids represent an interesting class of compounds that contribute greatly to the therapeutic arsenal in malaria treatment [59]. Grellier et al. [55] performed a series of* in vitro* and* in vivo* inhibition experiments on the aqueous root extracts of* C. sanguinolenta* on various strains of* Plasmodium falciparum* (*P. falciparum*) with varying degrees of resistance to chloroquine: the CQ-sensitive strain F32/Tanzania (IC_50_ CQ = 0.025 *μ*M) and the CQ-resistant strains FcB1/Colombia (IC_50_ CQ = 0.205 *μ*M) and FcR3/Gambia (IC_50_ CQ = 0.422 *μ*M). It was identified that the aqueous extracts significantly inhibited the* in vitro* growth of the* P. falciparum* strains irrespective of their degrees of resistance to chloroquine with IC_50_ ranging from 1 to 2 *μ*g/ml.* In vitro* testing of cryptolepine showed that cryptolepine, whether in the hydrochloride form or not, had IC_50_ values in the same range, 0.2–0.6 *μ*M, for the* P. falciparum strains* irrespective of the resistant levels [55]. Grellier and his team selected cryptolepine for further* in vivo* testing due to its impressive* in vitro* activity. It was identified that development of* P. vinckei petteri* and* P. berghei* parasites in mice was significantly inhibited by cryptolepine with the former demonstrating more sensitivity. Even with significant variability in the response to a single dose observed for both parasites in a single group of mice in three independent* in vivo* experiments, the results of this study strongly indicate the plants' inhibitory effect on* Plasmodium* species. Evidence provided by other independent studies indicates the antiplasmodial* in vitro* (IC_50_ = 114 nM, SI = 9, CQR) and* in vivo* (mice, ED_50_ < 50 mg/kg p.o. and ED_50_ = 10 mg/kg i.p.) properties of the* C. sanguinolenta* derived alkaloid, cryptolepine [17, 52, 60].

Paulo and his team [53] also investigated the* in vitro* antiplasmodial activity of the leaf and root extracts of* C. sanguinolenta* as well as the antiplasmodial effect of its alkaloids by testing them against the multidrug-resistant strain K1 and chloroquine-sensitive T996 clone of* P. falciparum*. All extracts were identified to inhibit 90% of* P. falciparum* K1 growth at concentrations below 23 *μ*g/ml with the root extracts demonstrating more activity than the leaf extracts. Ethanolic extracts of two out of three samples showed greater activity compared to their corresponding aqueous extracts [27]. Onyeibor et al. showed that cryptolepine and other derivative alkaloids from* C. sanguinolenta* inhibit hemozoin polymerization; this may in part explain the plants' antiplasmodial activity [61].

In a study conducted to characterize the drug-likeness properties of cryptolepine and also to determine whether a safe and novel antimalarial combination could be developed in combination with the artemisinin derivatives against late stage gametocytes of* P. falciparum* (NF54), cryptolepine exhibited promising synergistic interactions* in vitro* with artesunate, artemether, dihydroartemisinin, and amodiaquine. The combination of cryptolepine with chloroquine and lumefantrine showed an additive effect, whereas antagonism was observed with mefloquine in an isobologram analysis.* In vivo*, Rane's test in ICR mice infected with* Plasmodium berghei* NK-65 strains was used to build an isobologram. The isobologram built from cryptolepine-artesunate (1 : 1) and fractions of their ED_50_s using* in vivo* Rane's test in ICR mice infected with* Plasmodium berghei* NK-65 strains showed the combination to exhibit synergy with an experimental potency of 1.02 ± 0.02 mg/kg which was significantly lesser than the theoretical potency of 8.3 ± 0.31 mg/kg. In the study, it was shown that the aqueous root extract of* C. sanguinolenta* and its major alkaloid, cryptolepine, had minimal inhibitory effects on the late stage* Plasmodium falciparum* NF54 gametocytes. Toxicological analysis at all doses of cryptolepine and in combination with 4 mg/kg artesunate showed no significant acute toxicity with presentation of no morphological changes in the kidney, spleen, stomach, and liver tissues. These findings provide enough basis for cryptolepine's use as a potential lead compound for further development of antimalarial medication, alone or in combination with other antimalarials [62].

In a clinical trial involving 44 subjects with uncomplicated malaria, designed to test the efficacy of a teabag formulation of* C. sanguinolenta* containing 2.5 g root powder administered three times daily for five days, the formulation cleared* P. falciparum* parasitemia of 50% of the study subjects within three days and all study subjects by day 7. With the exception of hematological derangements following clinical manifestation of the disease, symptoms such as fever, chills, nausea, and vomiting resolved rapidly in 72 hours. The overall cure rate of the teabag formulation of* C. sanguinolenta* was 93.5% due to two cases of recrudescence on days 21 and 28 [63].

### 9.2. Antibacterial Activity

Various extracts of* C. sanguinolenta* have been extensively reported to possess antimicrobial properties against a variety of microbial species. The findings of Paulo et al. parallel those of Cimanga et al. in confirming the plant's activity against Gram-positive and Gram-negative bacteria, an activity which the authors attribute to the presence of cryptolepine, the main alkaloid in* C. sanguinolenta* [64, 65]. Boakye-Yiadom [15] showed that less than 50 mg/ml of aqueous extract causes below moderate antibacterial activity, a finding that is consistent with a later work performed by Paulo et al. [64]. It might be imperative that the ethanol extract is used if antibacterial activity is desired. Mills-Robertson et al. [43], in a comparative study of the ethanol, hot water, and cold water extracts of* C. sanguinolenta*, identified the ethanol extract to be more active against Gram-positive and Gram-negative bacteria than the other extracts. However, in a later study, the authors identified the chloroform extract as most active followed by the aqueous extract and then ethanol, opposing their earlier findings [42]. The antibacterial activity is highly attributed to the indole alkaloid cryptolepine, which has been shown to cause morphological changes and cellular breakdown in* Staphylococcus aureus* [43, 66]. However, the similar microbial activity possessed by other alkaloid derivatives from* C. sanguinolenta* indicates that the plants' antibacterial activity is not solely dependent on cryptolepine but on other alkaloids as well [64]. It is believed that the DNA intercalating and topoisomerase II inhibiting effects, as reported by Ansah et al. and others, may as well be the mechanism of action involved in antibacterial effects [64, 67–71].* C. sanguinolenta* has been found to be active against* Pseudomonas aeruginosa, Escherichia coli, Salmonella* Typhi*, Klebsiella pneumoniae*, and* Bacillus subtilis* [35, 72, 73]. Combination with other bactericidal plants such as* Crateva adansonii* in 1 : 2 or 2 : 1 can produce synergistic effects [35].

*Cryptolepis sanguinolenta* extracts were tested against the pan-sensitive H37Rv, the rifampicin-resistant TMC-331, and a wild strain of* Mycobacterium avium* isolated from a Ugandan patient. The total crude methanol extract showed the highest activity against H37Rv and TM-331 with complete clearance of quadrants at 50 mg/ml although it was not effective against wild strain* Mycobacterium avium* [26]. Gbedema et al. [74] reported the significant enhancement in amoxicillin's activity against* B. subtilis* in the presence of subinhibitory concentrations of* C. sanguinolenta*. In a study by Cimanga et al. [75], they identified that neocryptolepine and biscryptolepine, isolated from a hydroalcoholic extract of* C. sanguinolenta*, exhibited bacteriostatic effects rather than bactericidal effects on selected organisms. Neocryptolepine demonstrated greater inhibition of Gram-positive bacteria with weaker effects on Gram-negative ones. Biscryptolepine exhibited activity only against some Gram-positive bacteria but cryptoquindoline showed no activity against all the selected bacteria [75].

### 9.3. Anti-Inflammatory and Analgesic Activity

*Cryptolepis sanguinolenta* has been used ethnomedically in the treatment of various inflammatory conditions [21]. Research has shown that cryptolepine, the major alkaloid of the plant, inhibits* in vitro* nitric oxide production and DNA binding of nuclear factor-kappa B following inflammatory stimuli [76]. Olajide and his team identified that intraperitoneal administration of 10–40 mg/kg cryptolepine resulted in significant dose-dependent inhibition of the carrageenan-induced rat paw edema and carrageenan-induced pleurisy in rats. The alkaloid also dose-dependently caused analgesia and inhibited lipopolysaccharide-induced microvascular permeability in mice and writhing induced by intraperitoneal administration of acetic acid in mice. At the highest dose of 40 mg/kg, the* C. sanguinolenta* derived alkaloid exhibited significant anti-inflammatory and analgesic effect without inducing the formation of gastric lesions [76]. In a later study, Olajide and his team identified that* C. sanguinolenta*, at the doses used, produced dose-dependent inhibition of IL-1*β*-induced PGE_2_ release from SK-N-SH cells. Western blot experiments revealed that the extract at the doses used inhibited IL-1*β*-induced COX-2 and p38 expressions in these cells. This study provides evidence that* C. sanguinolenta* root extract inhibits the production of PGE_2_ in IL-1*β*-stimulated neuroblastoma cells through inhibition of COX-2 protein. The group suggested that the observed effects may be dependent on the inhibition of p38 MAP kinase activation [77]. In a study to elucidate the mechanism of action of* C. sanguinolenta* and its alkaloid cryptolepine in the inhibition of neuroinflammation, Olajide and his team showed that the plant extract and its derivative alkaloid both significantly inhibit TNF-*α*, IL-6, and PGE_2_ production in SK-N-SH cells and IL-1*β*-stimulated cells. Based on their findings, the team proposed that cryptolepine inhibits neuroinflammation through mechanisms involving inhibition of COX-2 and mPGES-1, probably mediated through NF-*κ*B and p38 signaling [77]. Bamgbose and Noamesi [10] had earlier demonstrated that cryptolepine, the major alkaloid of* C. sanguinolenta*, directly inhibits PGE_2_ without affecting prostaglandin production in the lungs. However, unlike Olajide and his team [76], Bamgbose and Noamesi [10] did not attribute the effect observed to the inhibition of COX, probably due to the little evidence available on the isoenzymes of COX at the time [78]. The anti-inflammatory activity of the methanol root extract of* C. sanguinolenta* has also been demonstrated by Odoh et al. [79] in two separate experiments to determine the extracts' effect in egg albumin, formalin, dextran, and carrageenan-induced paw edema in rats. The team identified that* C. sanguinolenta* at the doses administered significantly inhibited paw edema in a dose-dependent manner [79].

### 9.4. Antifungal Activity

In a study conducted, the indoloquinoline alkaloid of* C. sanguinolenta*, cryptolepine, inhibited different strains of* Saccharomyces cerevisiae* and* Candida albicans* at *μ*g/ml concentrations of 5–10, 10–20, 40–80, and 80–160 for* S. cerevisiae* NCPF 3139,* S. cerevisiae* NCPF 3178,* C. albicans* ATCC 10231, and* C. albicans* NCPF 3262, respectively. Time kill kinetics showed a reduction in viable count from 10^6^ to <10 cfu/ml in 4 hours for* C. albicans* ATCC at an exposure level of 320 *μ*g/ml. A 3 log cycle reduction was observed after exposing* S. cerevisiae* NCPF 3139 to 160 *μ*g/ml of cryptolepine. It was identified that exposure to biocidal concentrations resulted in extreme disturbance of surface structure, including partial and total collapse resulting in lysis of the cells [66]. Independent studies by Boakye-Yiadom [15], Agboke et al. [35], and Silva et al. [72] provide similar evidence on the plants' antifungal activity. In a study by Ekundayo and Ezeogu [73], both the methanol and the dichloromethane root extracts inhibited* C. albicans* growth with zones of inhibition of 8 mm and 10 mm, respectively. Agboke et al. [35] demonstrated the susceptibility of* Candida albicans* and* Aspergillus niger* to the crude extract of* C. sanguinolenta* using the agar diffusion method where the extract was able to inhibit the growth of both organisms at the same concentration of 12.5 mg/ml. Though a limitation of these studies is the absence of a positive control, their relevance is irrefutable.

### 9.5. Antiamoebic Activity

*Entamoeba histolytica* is among the most common pathogens responsible for diarrhea in the tropics and regions with poor sanitation [80]. Tona et al. [20] investigated the effect of the plant extract among other plants on* Entamoeba histolytica* strain isolated from patients with acute amoebic dysentery.* C. sanguinolenta* was shown to inhibit the growth of* E. histolytica* with a minimum inhibitory concentration of less than 7.81 *μ*g/ml [18]. The study therefore provides both scientific backing for the ethnomedical use of* C. sanguinolenta* in the treatment of intestinal amoebiasis and basic evidence to warrant further study to fully elucidate its antiamoebic effects.

### 9.6. Anticancer Activity

The anticancer activity of* C. sanguinolenta* has been mainly attributed to its major alkaloid, cryptolepine, although other studies have implicated other synthetic derivatives of some alkaloidal isolates of* C. sanguinolenta* [78, 81, 82]. An extensive review on the anticancer activity of* C. sanguinolenta* and its alkaloid, cryptolepine, has been published by Ansah and Mensah who proposed, based on recent evidence, that a close relationship exists between the plant's mechanism of cytotoxicity and its anti-inflammatory activity [78]. This review therefore will only look at a summary of it and other studies conducted after that period.

The major alkaloid of* C. sanguinolenta,* cryptolepine, has been shown to inhibit NF-*κ*B in various cells and also to induce cell cycle arrest and apoptosis in human lung adenocarcinoma A549 cells [83, 84]. In their review, Ansah and Mensah [78] in summary reported that the cytotoxicity and anti-inflammatory activity of* Cryptolepis*/cryptolepine are likely to be mediated by interference with NF-*κ*B activity leading to downregulation of inflammatory and antiapoptotic genes such as COX-2, iNOS, TNF-*α*, and* Bcl-2* genes. The inhibition of NF-*κ*B also leads to the upregulation of the proapoptotic genes p53, p21, Bax, caspase, and* cytochrome c* [78]. In an independent study, Olajide et al. [85] investigated whether the apoptotic-inducing effect of cryptolepine was mediated through the NF-*κ*B signaling pathway. The authors reported that cryptolepine dose-dependently inhibited A549 cell proliferation after 24 h of treatment with significant induction of caspase-3 and increase in relative luminescence in the cells. Protein analyses revealed that cryptolepine inhibited TNF-*α*-induced I*κ*B phosphorylation and NF-*κ*Bp65 nuclear translocation. Pretreatment with cryptolepine reduced the levels of* Bcl-2*, cyclin D1, survivin, XIAP, and cIAP in cells stimulated with TNF-*α*. The result of the study shows that cryptolepine downregulates the expression of antiapoptosis proteins and induces apoptosis in A549 lung carcinoma cells by interfering with NF-*κ*B signaling [85].

In a study by Pal and Katiyar [86], it was found that cryptolepine inhibits topoisomerase I and II activity with associated substantial DNA damage. Cryptolepine-induced DNA damage resulted in an increase in phosphorylation of ATM/ATR, BRCA1, Chk1/Chk2, and *γ*H2AX and also the activation of p53 signaling cascade, as previously reported by Zhu and Gooderham [84], including enhanced protein expressions of the cyclin-dependent kinases p16 and p21. The DNA damage also resulted in downregulation of cyclin-dependent kinases, cyclin D1, cyclin A, cyclin E, and proteins involved in cell division such as Cdc25a and Cdc25b, leading to cell cycle arrest at S-phase. Disruption of mitochondrial membrane potential and release of cytochrome c were also reported. These cryptolepine-induced changes in human nonmelanoma skin cancer cells resulted in a significant reduction in cell viability and colony formation and increase in apoptotic cell death [86].

### 9.7. Anxiogenic and Sedative Activity

Normally, agents that prolong phenobarbitone-induced sleeping time are expected to be anxiolytics in animal models. However, in a study, the aqueous extract of* C. sanguinolenta* reduced spontaneous locomotor activity and prolonged phenobarbitone sleeping time in mice but induced anxiogenic behavior in the same study. The extract decreased rearing and head dipping and caused an increase in nose poking in mice, which is consistent with anxiogenic behavior [87]. The anxiogenic and sedative effects of* C. sanguinolenta* may indicate the different mechanisms by which the extract exerts its effect. This variability in effect may be explained on the basis of the phytochemical composition of the plant, mostly its indole alkaloids, each of which may exert its effect via a different mechanism.

### 9.8. Antioxidant Activity

Cimanga and his team [88] investigated the effects of* C. sanguinolenta*-isolated alkaloids on xanthine oxidase and the production of superoxide anions. From the results of the study, it was shown that cryptoquindoline, quindoline, cryptolepine, neocryptolepine, and biscryptolepine were devoid of effects on xanthine oxidase activity with no recognizable activity against superoxide anion production. However, 11-hydroxycryptolepine was identified to exhibit significant inhibition of xanthine oxidase and the production of superoxide anions. With 11-hydroxycryptolepine having a distinctive hydroxyl group as opposed to the other alkaloids, it is suggested that the presence of a hydroxyl group is important for the inhibition of xanthine oxidase and the production of superoxide radicals [88].

### 9.9. Antidiabetic Activity

In a study to investigate the antidiabetic effect of* C. sanguinolenta*, the ethanolic extract was identified to significantly decrease the intestinal absorption and transport of glucose, resulting in decreased plasma glucose concentration. This effect was coupled with an observed decrease in fasting lipid cholesterol, triglycerides, and low-density lipoprotein (LDL) cholesterol levels and also an increase in high-density lipoprotein (HDL) cholesterol levels. Histopathological analysis showed an increase in the sizes of the Islet of Langerhans and altered pancreatic *β*-cell counts. The results of the study suggest the beneficial effects of* C. sanguinolenta* in reducing plasma glucose concentration and lipid levels and improving pancreatic *β*-cell function, the effects of which are very critical in the prevention of microvascular and macrovascular diseases in patients with diabetes mellitus [89].

In a murine diabetes model, cryptolepine significantly lowered plasma glucose levels. It was identified that the glucose lowering effect of cryptolepine led to significant reduction in plasma insulin concentration which is associated with evidence of improved insulin-mediated glucose disposal. The authors also reported a cryptolepine-mediated increase in glucose uptake by the 3T3-L1 cells [24]. Following their earlier discovery of the antihyperglycemic effect of cryptolepine [22], Bierer and his colleagues measured the antihyperglycemic effects of a series of synthetic heterosubstituted cryptolepine analogs in a noninsulin-dependent diabetes mellitus (NIDDM) model. From their study, Bierer and his colleagues were able to generate the first structure-bioactivity study about the cryptolepine nucleus [23].

To evaluate the relevance of cryptolepine, the major alkaloid of* C. sanguinolenta* in the long-term management of diabetes mellitus, Ameyaw et al. [90] examined the effect of the alkaloid in alloxan-induced diabetes. Cryptolepine treatment significantly (*p* ≤ 0.001) reduced fasting blood glucose and body weight and inhibited the latency to withdrawal from pain stimulus. The authors reported a decrease in plasma urea levels and elevation in plasma creatinine associated with diabetes mellitus. Similar to the reports by Cimanga et al. [88], it was identified that cryptolepine reversed diabetes-associated elevation of plasma cholesterol, triglycerides, and low-density lipoproteins, with reduction in high-density lipoproteins. Cryptolepine exhibited dose-dependent regeneration of *β*-islet cells; however, it could not repair degenerated liver and kidney tissues. The alkaloid was realized to dose-dependently worsen diabetes-mellitus-associated reduced sperm quality. Evidence from this study depicts cryptolepine's ability to inhibit hyperglycemia, weight loss, cold allodynia, neuropathic pain, and hyperlipidemia associated with diabetes mellitus. Regeneration of pancreatic *β*-islet cells by the alkaloid is very important in the management of diabetes mellitus. Cryptolepine, however, does not improve liver and kidney damage [90].

### 9.10. Antifertility and Reproductive Toxicity

The extract of* C. sanguinolenta* has been shown to reduce male and female fertility, terminate pregnancy when given before organogenesis, induce fetal mortality, and cause intrauterine growth restriction in animal studies. Fetal death may be a result of cell cycle arrest at the G_1_ phase with subsequent irreparable embryonic cell damage following the administration of the extract [91]. If this occurs at the very early stages of conception, resorption may occur, which may lead to reduced fertility.

In a reproductive toxicological study in male mice,* C. sanguinolenta* extract at all doses decreased male fertility, which was also reflected in the decreased female fertility index, with no significant differences in postimplantation losses between the observed treated groups [91]. The team reported a reduction in the mean weight of the left caudal epididymis and the testes following treatment with the extract, although the result was not significant (*p* > 0.05) compared to the control. However, treatment with the extract resulted in a significant (*p* < 0.05) reduction in sperm numbers. Cryptolepine, the major alkaloid, via its antimuscarinic effect, *α*-adrenoceptor antagonism, and cytotoxicity, may in part be responsible for the decrease in fertility in the male rodent population treated with* C. sanguinolenta* [68, 69, 92]. The genotoxic effect of the extract observed at high doses depicts the presence of a weak genotoxic principle within the plant. Administration of* C. sanguinolenta* extract to pregnant mice before organogenesis and throughout the gestational period resulted in higher incidences of intrauterine growth restriction and 40–46% incidence of abortion compared to 0% in the control group. Although no anatomical malformations in the limbs, spine, and palate were observed, mortality in offspring born to* C. sanguinolenta* extract treated mice was significantly higher: 12% compared to the 0.5% offspring mortality in the control.

The antifertility effect of* C. sanguinolenta* has also been demonstrated by Ameyaw et al. [90] and Akhigbe et al. [89]. These studies provide evidence that supports the hypothesis that* C. sanguinolenta* extract significantly suppresses sperm count. The ethanol extract of the plant has been reported to significantly decrease testosterone levels with an associated rise in the levels of luteinizing hormones. However, the extract appeared not to affect the levels of follicle stimulating hormones. Histomorphological analysis showed no alterations in the testicular tissues of rats treated with 50 and 150 mg/kg of the extract; however, mild distortion of the seminiferous tubules at a dose of 250 mg/kg was observed [89]. Similar to the report by Ansah and his team [91], Akhigbe et al. [89] also reported a nonsignificant change in testicular morphological parameters such as testicular weight, length, and diameter.

The ability of* C. sanguinolenta* to reduce fertility has been shown to be consistent with COX-2 inhibitors-mediated fertility reduction [91]. The antifertility effect may therefore be a result of inhibition of COX-2, which the extract has already been shown to inhibit in its anti-inflammatory activity. Though reproductive studies have not been conducted in humans, the results of these studies indicate the potential contraindication of* C. sanguinolenta* or any preparation containing* C. sanguinolenta* in pregnant women and women of reproductive age who want to conceive.

### 9.11. Ulcerogenicity

*Cryptolepis sanguinolenta* has been shown to increase basal acid secretion as well as histamine-induced gastric acid secretion. In a study by Ajayi et al. [93], the ethanol extract of* C. sanguinolenta* was identified to dose-dependently increase the number and sizes of gastric parietal cells, explaining the increase in gastric acid secretion after the administration of the extract, increasing the risk of gastric ulceration. However, Olajide and his team [11] employed a high dose of cryptolepine for analgesia and for preventing inflammation in mice without inducing gastric ulceration. This may imply that the gastric secretagogue action of the extract may not be a resultant effect of its major alkaloid, cryptolepine. Since this association is inconclusive, further studies need to be done to properly understand the effects of* C. sanguinolenta* and its derived alkaloids in the gastric mucosa. The study by Ajayi et al. [93] also demonstrated the appetite and weight gain stimulating effect of* C. sanguinolenta*.

### 9.12. Enzyme Activity

Treatment with cryptolepine, the major alkaloid of* C. sanguinolenta*, resulted in a further increase in the elevated levels of plasma aspartate transaminase associated with induction of diabetes mellitus in Sprague-Dawley rats. However, no significant effects on elevated levels of alanine transaminase and gamma-glutamyl transferase were observed [90]. However, in the absence of any disease, administration of the plant extract resulted in no changes in serum transaminases, suggesting the plants' limited effect on the liver [88]. Concurrent administration of artesunate with* C. sanguinolenta* extract reduces the plasma concentration of artesunate leading to subtherapeutic drug levels and, ultimately, drug resistance. Coadministration of the two has been shown to induce the cytochrome P450 enzyme isoforms CYP1A and CYP2B1, leading to increased metabolism of artesunate. The extract, however, did not significantly affect the levels CYP2E1 in the presence of artesunate [69].

## 10. Pharmacokinetics

In vitro pharmacokinetic assays of cryptolepine in rat and human plasma demonstrated high passive permeability, low human* p*-glycoprotein efflux potential, good metabolic stability, and moderate protein binding by cryptolepine. Preliminary incubation in human and rat hepatocytes showed low to moderate hepatic extraction with nine metabolites identified in the hepatocytes of both organisms. The metabolites (Table 5) were proposed to have resulted from metabolic pathways involving oxidation (M2–M9) and glucuronidation (M1, M2, M4, M8, and M9). The metabolites M2, M6, and M9 were also identified in the rat urine and the M6 metabolite was also identified in rat plasma. M1 was only identified in human hepatocytes while the M8 and M9 metabolites were only identified in rat hepatocytes. These hepatocyte-associated metabolites together with enzyme phenotyping assay suggest the possible involvement of both cytosolic and microsomal liver enzymes in the metabolism of cryptolepine in the rat and human hepatocytes. Aldehyde oxidase, UDP-glucuronyltransferase, and the cytochrome P450 enzyme system may be among the enzymes implicated. In vivo rat pharmacokinetic profile of cryptolepine showed very high clearance and volume of distribution, a moderate half-life, low oral exposure, early time to peak, and a low peak concentration. Elimination was faster and systemic exposure to cryptolepine was low to moderate in rats with unchanged excretion of cryptolepine in the urine less than 0.1% of the administered dose. This indicates metabolism, unchanged drug, and/or biliary excretion as the main clearance pathway(s). Elimination of cryptolepine was faster with less than 0.1% of the administered dose excreted unchanged in urine [62].

## 11. Toxicological Assessment

In an* in vitro* study, the toxicological profiles of* C. sanguinolenta* and its major alkaloid, cryptolepine, were established using the Chinese hamster lung fibroblast (V79-MZ) cell line, human colon adenocarcinoma (HCT116) cell line, human ovary adenocarcinoma (SKOV3) cell line, and the human breast adenocarcinoma MCF7 and MDA MB 361 cell lines. The plant and its alkaloid both caused a dose- and time-dependent reduction in viability of the V79 cell line. The DNA histograms from flow cytometry analysis indicated that treatment with 5 *μ*g/ml* C. sanguinolenta* extract or 0.5 *μ*M cryptolepine for 24 hours did not appear to have any significant effect on the cell cycle distribution. However, after treatment with 50 *μ*g/ml* C. sanguinolenta* extract or 5 *μ*M cryptolepine, a gradual accumulation of sub-G1 cell population began to emerge in a dose-dependent fashion, accounting for about 55% of the population with these sub-G1 cells most likely being apoptotic [68]. A significantly different result, that is, 30% cell death, was observed with the Trypan Blue test, a finding which the authors attributed to the fact that a significant number of the dying cells excluded Trypan Blue and were probably early apoptotic. The authors then reported strikingly similar results on* C. sanguinolenta* and cryptolepine effects on the cycle distribution following a confirmatory test for apoptosis using light microscopy and Diff-Quick®. Treatment with the extract and the alkaloid profoundly inhibited the growth of V79 cells and almost all the organ-specific human cancer cell lines. In a V79 cell mutation assay (hprt gene),* C. sanguinolenta* extract only induced mutation at the highest dose employed with mutation frequency of approximately 4 and 38 mutant clones per 106 cells for control and the extract, respectively. Cryptolepine however at 0.5–5.0 *μ*M was not mutagenic [68]. In his Ph.D. thesis, Mensah identified that* Cryptolepis sanguinolenta* at a dose beyond 100 mg/kg, administered orally, progressively induced an apoptotic-like cell death in the kidneys of mice [94]. These results indicate the cytotoxicity of* C. sanguinolenta* and its alkaloid cryptolepine with both probably weak mammalian mutagens and/or clastogens [68].

With the exception of a reduction in mean cell volume and an accompanied increase in mean cell hemoglobin concentration due to unchanged hemoglobin, all other hematological parameters and serum transaminases were unaffected following treatment of rats with* C. sanguinolenta* for two weeks [88]. A study by Ajayi et al. [93] also demonstrated the stability of most hematological parameters against treatment with the ethanolic extract of* C. sanguinolenta*. Both daily administration of* C. sanguinolenta* extract for two weeks and 30-minute administration prior to pentobarbitone administration dose-dependently prolonged rat sleeping time. The effect as explained by the authors is CNS-related as opposed to being enzymatic due to a decrease in locomotor activity observed at a dose of 500 mg/kg [88]. In contrast to their earlier study, Ansah and his team [30] identified a dose-dependent increase in platelet and granulocyte numbers following two weeks of administration of* C. sanguinolenta*. Similarly, the work done by Ajayi and his team [93] demonstrated the extracts' selective stimulatory effect on bone marrow, evidenced as a selective rise in platelet and white blood cells. Administration of 2000 mg/kg of the extract was associated with marginal enlargement of the liver and kidney; however, this finding did not correlate with findings from biochemical and histopathological studies, which showed no changes in the renal and hepatobiliary systems. From these studies, it may be suggested that the root extract of* C. sanguinolenta* is generally safe at doses below 500 mg/kg and caution should be taken in administering doses above 500 mg/kg [30].

## 12. Conclusion

There have been documented folkloric uses of* Cryptolepis sanguinolenta* in the literature. Also, there has been a sizeable amount of research to back these diverse folkloric benefits of this plant. However, there is a significant gap remaining to be filled concerning research into this plant which holds importance in medicine.

Although there have been data pointing to the toxic potential of the plant, it still serves as a source of potential agent(s) which hold prospects in therapy, spanning from the antimalarial benefits to the possible application in cancer chemotherapy.

We therefore recommend further studies on* C. sanguinolenta.* This is informed by the global need for more effective, however less toxic, therapeutic agents. Future studies on* C. sanguinolenta* can screen for possible chemotherapeutic potential as well as anti-inflammatory and analgesic activity of the extract and its bioactive constituents, such as cryptolepine, as potential therapy in humans.

## Figures and Tables

**Table 1:** Ethnopharmacological use of *Cryptolepis sanguinolenta*.

Part	Value	Reference(s)
Root	Fever, upper respiratory and urinary tract infections, septicemia	[15–17]
Not specified	Respiratory diseases, enteric diseases	[2–4]
Not specified	Insomnia	[18]
Fresh aerial parts	Diarrhea	[19, 20]
Not specified	Amoebiasis	[20]
Roots and leaves	Hypertension, inflammation, pyrexia, malaria	[21]
Not specified	Diabetes	[22–24]
Root	Stomach and intestinal disorders	[6, 25]
Root	Tuberculosis	[26]
Root	Hepatitis, wounds	[27]

**Table 2:** Vernacular names of *Cryptolepis sanguinolenta*.

Number	People	Vernacular name	Reference(s)
(1)	Ghana	Ghana Quinine	[30, 31]
(2)	Bantu	K*ɔ*li mekari	[32]
(3)	Guans	Nurubima	[31]
(4)	Ewe	Kadze	[31]
(5)	Twi	Nibima	[31, 33]
(6)	Yoruba	Paran pupa	[7]
(7)	Hausa	Gangnamau	[34, 35]

**Table 3:** Mean dimensions of the leaf, seed, petiole, and pod indices of *C. sanguinolenta* (adapted from Ameyaw [31]).

Location	Leaf length (cm)	Leaf petiole length (cm)	Seed length (cm)	Pod length (cm)	Seed/hair length (cm)
Pepease	7.9 ± 1.0483^*∗∗∗*^	0.9 ± 0.1545^*∗*^	0.8 ± 0.18^*∗*^	18.6 ± 3.5^*∗∗*^	5.3 ± 0.81^*∗*^
Mamfe	6.8 ± 1.0131^*∗∗*^	1.4 ± 0.2429^*∗∗∗*^	1.0 ± 0.23^*∗∗∗*^	15.9 ± 2.44^*∗*^	6.0 ± 0.81^*∗∗∗*^
Abonse	6.1 ± 0.6254^*∗*^	1.1 ± 0.3172^*∗∗*^	0.9 ± 0.17^*∗∗*^	18.7 ± 2.74^*∗∗∗*^	5.4 ± 0.96^*∗∗*^

^*∗∗∗*^ *p* < 0.001; ^*∗∗*^*p* < 0.01; ^*∗*^*p* < 0.05.

**Table 4:** Mean dimensions of the fiber length, vessel element length, and prismatic crystal length (adapted from Ameyaw [31]).

Location	Fiber length (*μ*m)	Vessel length (*μ*m)	Prismatic crystal length (*μ*m)
Pepease	52.5 ± 0.53^*∗∗∗*^	34.5 ± 3.541^*∗∗∗*^	2.25 ± 0.032^*∗∗∗*^
Mamfe	37.0 ± 0.43^*∗*^	32.6 ± 3.230^*∗∗*^	2.25 ± 0.032^*∗∗∗*^
Abonse	40.6 ± 0.27^*∗∗*^	30.2 ± 2.862^*∗*^	2.21 ± 0.031^*∗∗∗*^

^*∗∗∗*^ *p* < 0.001; ^*∗∗*^*p* < 0.01; ^*∗*^*p* < 0.05.

**Table 5:** Liquid chromatography-mass spectrometry data for cryptolepine and the proposed metabolites (adapted from Donkor [62]).

Metabolite/parent compound	Retention time (min)	The proposed MH+ formula	Measured *m*/*z*
M1	12.9	C_22_H_21_N_2_O_6_	409.1394
M2	14.8	C_22_H_21_N_2_O_7_	425.1344
M3	14.8	C_16_H_15_N_2_O_2_	267.1129
M4	15.8	C_22_H_21_N_2_O_7_	425.1343
M5	17.1	C_16_H_15_N_2_O_3_	283.1078
M6	18.1	C_16_H_13_N_2_O	249.1023
M7	18.6	C_16_H_13_N_2_O	249.1022
M8	18.7	C_22_H_21_N_2_O_8_	441.1294
M9	19.2	C_22_H_21_N_2_O_8_	441.1292
Cryptolepine	18.5	C_16_H_13_N_2_	233.1073
